# Homocystinuria presenting with cerebral venous thrombosis: a case report highlighting progressive thrombosis

**DOI:** 10.3389/fcvm.2026.1825770

**Published:** 2026-06-19

**Authors:** Xiaomin Cui, Jinjing Chen, Jing Wang, Dezhi Qiu, Xianli Zhang

**Affiliations:** 1 Department of Surgical Intensive Care Unit, Children's Hospital of Nanjing Medical University, Nanjing, Jiangsu, China; 2 Department of Neurosurgery, Affiliated Hospital of Integrated Traditional Chinese and Western Medicine, Nanjing University of Chinese Medicine, Nanjing, Jiangsu, China; 3 Department of Neurosurgery, Children's Hospital of Nanjing Medical University, Nanjing, Jiangsu, China

**Keywords:** anticoagulant therapy, cerebral venous sinus thrombosis, Cystathionineβ-synthase deficiency, genetic testing, homocystinuria, thromboembolic events

## Abstract

**Background:**

Homocystinuria is a hereditary metabolic disorder primarily caused by defects in enzymes involved in methionine metabolism, resulting in excessive accumulation of homocysteine and its metabolites in the blood and urine. Cerebral venous sinus thrombosis (CVST), premature atherosclerosis, and other thromboembolic events are among the most serious clinical manifestations of homocystinuria.

**Case presentation:**

We report a 13-year-old boy who initially presented with headache, followed by progressive disturbance of consciousness and status epilepticus. Cranial magnetic resonance imaging (MRI) revealed superior sagittal sinus thrombosis. He was transferred to a tertiary hospital, where he underwent emergency thrombus aspiration under digital subtraction angiography (DSA) guidance and received low-molecular-weight heparin (LMWH) anticoagulation. Although endovascular aspiration combined with LMWH controlled the seizures, his venous thrombosis continued to progress. He subsequently developed lower-extremity deep vein thrombosis, acute pulmonary embolism, and ventricular fibrillation. Clinical biochemical evaluation and genetic testing confirmed classic homocystinuria. Targeted therapy with warfarin, aspirin, vitamin B6, folic acid, and betaine resulted in a favorable prognosis.

**Conclusion:**

Early etiological screening, including genetic testing, should be prioritized in young patients with unexplained or recurrent thrombosis to optimize treatment and prognosis. Rational use of anticoagulants combined with targeted metabolic therapy (vitamin B6, folic acid, betaine) and antiplatelet therapy is critical for improving outcomes in such patients.

## Introduction

Cystathionineβ-Synthase (CBS) deficiency, also referred to as classic homocystinuria, is a rare autosomal recessive disorder and represents the most prevalent congenital defect in sulfur amino acid metabolism (1). This disorder is characterized by impaired cystathionine synthesis, leading to the accumulation of homocysteine. Clinically, patients typically manifest a wide spectrum of manifestations between preschool age and adolescence, including intellectual disability, motor developmental delay, myopia, lens dislocation, thrombotic and cerebrovascular events, premature atherosclerosis, and a Marfan-like phenotype (2).

Here, we report a pediatric case complicated by intracranial venous sinus thrombosis, multiple atherosclerotic lesions, hypertension, and thromboembolic events. Upon admission, differential diagnoses were performed to rule out thrombophilia, Behcet's disease, systemic lupus erythematosus, malignancy, and other autoimmune disorders.Genetic testing confirmed the diagnosis of CBS deficiency. During the acute phase of pulmonary embolism and deep vein thrombosis progression, LMWH exhibited suboptimal anticoagulant efficacy, which was presumably associated with the patient's reduced antithrombin activity. Emergency thrombolysis with urokinase, followed by sequential anticoagulant therapy with unfractionated heparin and subsequent warfarin maintenance, achieved favorable efficacy in controlling the patient's overall condition. Therefore, we report this case to highlight that, amidst a broad and variable clinical presentation, establishing the etiological diagnosis of CBS deficiency is crucial for successful management. Furthermore, anticoagulation therapy should be individualized based on a comprehensive risk-benefit assessment.

## Case report

A 13-year-old boy presented with progressively worsening headaches over the past month, accompanied by left upper limb weakness and emotional instability. During hospitalization at a local hospital, he experienced multiple seizures followed by a rapid decline in level of consciousness. Cranial MRI, magnetic resonance angiography (MRA), and magnetic resonance venography (MRV) revealed superior sagittal sinus thrombosis (Figure 1A) with edema of the bilateral frontal and parietal lobes. On diffusion-weighted imaging (DWI), the edematous regions showed mild to moderate hyperintensity along the white matter tracts (Figure 2A). Corresponding T2-weighted fluid-attenuated inversion recovery sequences demonstrated concordant mild hyperintensity in the same distribution (Figure 2B). Note that, in the absence of apparent diffusion coefficient maps, T2 shine-through cannot be excluded as a cause of the DWI signal changes. Based on the MRV findings, the edema was considered predominantly vasogenic, although focal cytotoxic edema secondary to venous congestion could not be ruled out. Ambulatory blood pressure monitoring showed a 24 h mean systolic pressure of 155 mmHg and a mean diastolic pressure of 93 mmHg. Despite treatment with phenobarbital for sedation and nifedipine for blood pressure control, convulsive episodes recurred repeatedly. The patient was subsequently transferred to the Children's Hospital of Nanjing Medical University for further comprehensive management.

**Figure 1:**
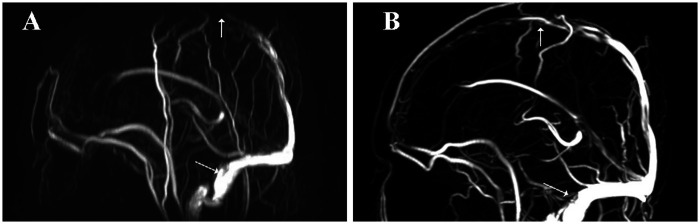
Utility of pre- and postoperative TOF MRV comparison. **(A)** Preoperative MRV shows a slender superior sagittal sinus (solid arrow) and left sigmoid sinus(dashed arrow) with segmental non-visualization. **(B)** Postoperative MRV demonstrates mild improvement, with residual focal filling defects in the anterior superior sagittal sinus (solid arrow) and left sigmoid sinus (dashed arrow).

**Figure 2:**
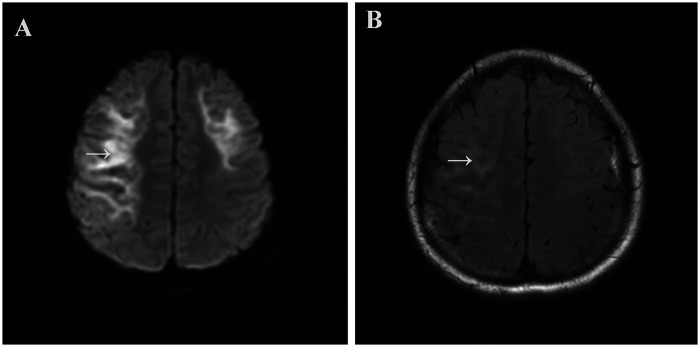
Axial brain MRI scans. **(A)** DWI shows mild-to-moderate hyperintensity along bilateral white matter tracts, with right predominance (solid arrow). **(B)** Corresponding T2-FLAIR sequence reveals concordant mild hyperintensity in the affected white matter tracts (solid arrow).

This 13-year-old Han Chinese boy had no positive family history of hereditary or congenital diseases. His father presented with recurrent oral ulcers, and his mother was physically healthy without chronic illnesses. The patient had normal intellectual function and a confirmed diagnosis of high myopia, with an unremarkable personal medical history. He was 170 cm tall, weighed 70 kg, habitually consumed greasy foods. Upon hospital admission, the patient presented with confusion, incoherent speech, and diminished muscle strength in the left upper extremity. As his disturbance of consciousness progressed rapidly, emergency cerebral angiography combined with mechanical thrombus aspiration was performed promptly. DSA showed complete occlusion at the distal right internal carotid artery (C1 segment), with no antegrade flow beyond the occlusion. The left internal carotid artery (C2 segment) had diffuse luminal stenosis and markedly reduced antegrade flow. Collateral compensation was observed via the bilateral posterior communicating arteries, where the vertebral arteries perfused the anterior cerebral circulation. During the venous phase of vertebral artery angiography, the anterior two-thirds of the superior sagittal sinus showed absent contrast enhancement, indicative of venous sinus thrombosis. Subsequent thrombus aspiration restored contrast opacification in this segment.Postoperatively, the patient began anticoagulation therapy with subcutaneous nadroparin calcium at 91 IU/kg every 12 h, and antiepileptic therapy with oral levetiracetam 0.5 g every 12 h.

Postoperative seizures resolved completely. Routine laboratory tests with diagnostic value were largely unremarkable: routine blood counts and infection markers were normal, lupus anticoagulant and antiphospholipid antibodies were negative, and Human Leukocyte Antigen-B51 Genotyping was unremarkable. D-dimer was elevated at 2,774 ng/mL, antithrombin III activity was markedly reduced at 33%, and neuron-specific enolase was elevated at 70.20 ng/mL, whereas tumor markers were normal.Further coagulation studies revealed markedly elevated von Willebrand factor activity (320.9%) and factor VIII activity (336.0%); protein C and protein S were normal. Thrombomodulin was normal at 7.7 TI/mL. Thrombin-antithrombin III complex was 29.4 ng/mL, plasmin-α2-plasmin inhibitor comple was 2.746 μg/mL, and tissue-type plasminogen activator-inhibitor complex was 29.6 ng/mL (Table 1). Extremity vascular ultrasonography revealed irregular intima-media thickening and wall roughness in the right brachial artery and bilateral femoral arteries, along with superficial venous thrombosis in the right cubital fossa and anteromedial left upper arm; no abnormalities were detected in the main venous trunks of both lower extremities. Whole-exome sequencing was performed for trio-based gene analysis.

**Table 1:** Lab measurement and test result.

Inspection items	Value	Reference range	Unite	Inspection items	Value	Reference range	Unite
WBC	15.57	4.1–11.0	10^9^/L	IgG	9.47	8.27–14.17	g/L
RBC	4.39	4.5–5.9	10^12^/L	IgG4	2,380	49–1,985	mg/L
Hb	151	129–172	g/L	IgM	0.36	1.22–2.56	g/L
HCT	44.8	39–51	%	lgA	2.9	0.86–1.92	g/L
MCV	102	80–100	fL	C3	0.801	0.88–2.01	g/L
RDW-SD	52.0	37–47	%	C4	0.213	0.16–0.47	g/L
PLT	236	150–407	10^9^/L	ASO	191	0–200	IU/mL
TBIL	12.4	3.4–17.1	umol/L	RF	<9.25	0–15.9	IU/mL
IBIL	7.1	1.7–13.7	umol/L	A-CCP	<8	≤17	U/mL
SUA	236	208–428	umol/L	LR	0.85	–	<1.2
LDH	319	120–290	U/L	ANA	<4.0	<32	AU/mL
PT	19.3	10–14	s	A-dsDNA	negative	<1:10	-
INR	1.69	0.86–0.63	–	aCL	<5.0	<16	AU/mL
APTT	46.6	20–45	s	Anti-β2GPI	<2.0	<16	AU/mL
Fib	2.47	1.8–4	g/L	A-ENAs	all negative	–	–
TT	13.1	12–21	s	PR3 IgG	<2.0	<16	AU/mL
D-Dimer	2,774	≤280	ng/L	MPO IgG	1.5	<16	AU/mL
AT-III	33	83–128	%	p-ANCA IgG	negative	<1:10	–
vWF Activity	320.9	49.5–187	%	c-ANCA IgG	negative	<1:10	–
Factor VIII	336.0	70–150	%	blood type	O,Rh positive	–	–
Factor XII	33.6	50–120	%	HIV Ab	0.194	<1	COI
Protein C	99.0	70–140	%	TPHA	0.076	<1	COI
Protein S	157.1	63.5–149	%	UOB	++	negative	–
TM	7.7	3.8–13.3	TU/mL	UP	+-	negative	–
TAT complex	29.4	0–4	ng/mL	URBC	141.6	0–10	/uL
PIC	2.746	0–0.8	Ug/mL	FC	221.3	0–75	ug/g
t-PAIC	29.6	0–17	ng/mL	HLA-B51	negative	–	–
hs-CRP	10.71	0–10	mg/L	PCT	0.045	<0.05	ng/mL
ESR	16	≤20	mm/h	NSE	70.2	0–16.3	ng/mL

WBC, white blood cell count; RBC, red blood cell count; Hb, hemoglobin; HCT, hematocrit; MCV, mean corpuscular volume; RDW-SD, red blood cell distribution width—standard deviation; PLT, platelet count; TBIL, total bilirubin; IBIL, indirect bilirubin; SUA, serum uric acid; LDH, lactate dehydrogenase; PT, prothrombin time; INR, international normalized ratio; APTT, activated partial thromboplastin time; Fib, fibrinogen; TT, thrombin time; D-Dimer, D-dimer; AT-III, antithrombin III; vWF Activity, von Willebrand factor activity; Factor VIII, factor VIII; Factor XII, factor XII; Protein C, protein C; Protein S, protein S; TM, thrombomodulin; TAT complex, thrombin-antithrombin complex; PIC, plasmin-α2-plasmin inhibitor complex; t-PAIC, tissue plasminogen activator-inhibitor complex; hs-CRP, high-sensitivity C-reactive protein; ESR, erythrocyte sedimentation rate; IgG, immunoglobulin G; IgG4, immunoglobulin G4; IgM, immunoglobulin M; IgA, immunoglobulin A; C3, complement component 3; C4, complement component 4; ASO, antistreptolysin O; RF, rheumatoid factor; A-CCP, anti-cyclic citrullinated peptide antibody; LR, lupus anticoagulant ratio; ANA, antinuclear antibody; A-dsDNA, anti-double-stranded DNA antibody; aCL, anticardiolipin antibody; Anti-β2GPI, anti-beta-2 glycoprotein I antibody; A-ENAs, anti-extractable nuclear antigens (profile); PR3 IgG, anti-proteinase 3 antibody IgG; MPO IgG, anti-myeloperoxidase antibody IgG; p-ANCA IgG, perinuclear anti-neutrophil cytoplasmic antibody IgG; c-ANCA IgG, cytoplasmic anti-neutrophil cytoplasmic antibody IgG; blood type, ABO blood type and Rh(D) status; HIV Ab, human immunodeficiency virus antibody; TPHA, Treponema pallidum hemagglutination assay; UOB, urine occult blood; UP, urine protein; URBC, urine red blood cell count; FC, fecal calprotectin; HLA-B51, human leukocyte antigen B51; PCT, procalcitonin; NSE, neuron-specific enolase.

In the early morning of postoperative day 4, the patient developed recurrent seizures followed by ventricular fibrillation. After successful cardiopulmonary resuscitation, bedside ultrasonography strongly suggested pulmonary embolism. Urokinase thrombolysis was administered immediately. Given severe cardiac dysfunction, extracorporeal membrane oxygenation (ECMO) was initiated for salvage therapy. Given the need for rapid onset, short half-life, and titratability in the setting of ECMO, along with the potential for renal impairment after resuscitation, the anticoagulation was switched from LMWH to continuous intravenous infusion of unfractionated heparin (UFH), guided by ACT monitoring (target 180–220 s) (3).

After 43 h, the patient's cardiac function recovered, and ECMO was discontinued. Intravenous heparin was discontinued, and subcutaneous injection of nadroparin calcium 91 IU/kg every 12 h was continued in combination with oral aspirin 100 mg once daily. After 3 days, the ventilator was discontinued. Enhanced Computer Tomography revealed: multiple thrombi within bilateral lower pulmonary artery branches; marked narrowing of the left external iliac artery compared to the contralateral side; thickened, irregular walls with distal narrowing of the superior mesenteric artery and bilateral common femoral arteries; thrombus formation in the left common iliac vein, distal external iliac vein, and bilateral common femoral veins. Follow-up Cranial MRA and MRV demonstrated a decrease in filling defects within the superior sagittal sinus compared with the prior examination, with residual focal defects in the anterior superior sagittal sinus and left sigmoid sinus (Figure 1B). Distal branches of the right middle cerebral artery appeared reduced in number (Figure 3A). The bilateral internal carotid arteries were narrowed with poorly visualized segments, more pronounced on the right (Figure 3B). Multiple abnormal signals were observed in the right frontal lobe, right frontotemporal region, subcortical area of the left frontotemporal lobe, and the left centrum semiovale, consistent with thrombosis of the bilateral superficial frontotemporal cortical veins and the superior sagittal sinus, accompanied by partial hemorrhagic infarction.Following the suboptimal response to low molecular weight heparin in the setting of significantly reduced antithrombin activity (33%; normal range: 83%–128%), the patient was transitioned to warfarin. Aspirin was continued concurrently to address the multiple atherosclerotic lesions.

**Figure 3:**
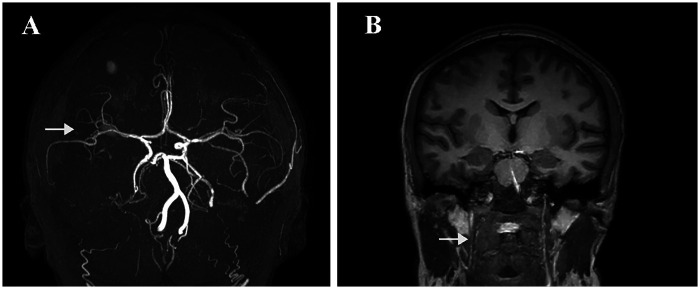
Utility of TOF MRA. **(A)** MRA shows a paucity of distal branches in the right middle cerebral artery (solid arrow). **(B)** MRA reveals attenuation of the bilateral internal carotid arteries, with segmental signal loss more pronounced on the right (solid arrow).

The patient's mental status improved during medication. Whole-exome sequencing for trio-based gene analysis identified two heterozygous pathogenic variants in the CBS gene:1.c.1111G > A (p.V371M), classified as pathogenic (P), maternally inherited;2.c.502G > A (p.V168M), classified as likely pathogenic (LP), paternally inherited. A diagnosis of CBS deficiency was made. Laboratory tests showed markedly elevated serum homocysteine (247.8 μmol/L) and plasma methionine (83.27 μmol/L; reference range: 8.95–39.22 μmol/L). Accordingly, treatment was initiated with vitamin B6 (100 mg daily in three divided doses) and folic acid (5 mg daily). Due to rapid thrombus progression and significantly elevated homocysteine levels, betaine (500 mg three times daily) was administered to the pediatric patient owing to the yet undetermined responsiveness to vitamin B6.

At the 2-week follow-up, bilateral lower extremity vascular ultrasonography demonstrated improved focal wall roughness of the bilateral femoral arteries; no significant stenosis or wall thickening was detected in the remaining arteries, veins, or their branches. Cranial MRI revealed reduced extent of abnormal signals and perilesional edema in the bilateral frontal and parietal lobes. Antithrombin III activity recovered to 66%. The patient's headaches and seizures resolved without recurrence, and he was discharged on maintenance medications. At the six-week follow-up, the patient's homocysteine and methionine levels were 24.5 μmol/L and 24.94 μmol/L, respectively. Five months after discharge, the patient had good neurological recovery, with mRS score improving from 4 at admission to 0. He remained alert and free of headache, seizures, and facial palsy. The lameness resolved completely, and he had good exercise tolerance. Laboratory follow-up showed elevated homocysteine (37.6 μmol/L) and methionine (30.31 μmol/L). The Wechsler Intelligence Scale for Children yielded a score of 70. According to the European Stroke Organisation and American Heart Association/American Stroke Association guidelines (4), anticoagulation for cerebral venous sinus thrombosis is recommended for 3–6 months. Considering the time needed for endothelial repair, we discontinued warfarin and aspirin at the five-month follow-up. The patient continued oral levetiracetam, vitamin B6, folic acid, and betaine with good adherence. Long-term metabolic monitoring and regular clinical follow-up are essential to reduce the risks of thrombus recurrence and neurocognitive dysfunction.

## Disscussion

Cystathionineβ-synthase (CBS) deficiency is a rare autosomal recessive disorder with an estimated incidence of approximately 1 in 200,000 in the United States (5). Severe cases usually present in childhood with lens dislocation, learning difficulties, and skeletal abnormalities (6). Thromboembolic events are relatively uncommon during adolescence in patients with CBS deficiency.Cerebral venous sinus thrombosis (CVST) accounts for 0.5%–1.0% of all strokes (7). In the general population, the annual incidence of CVST is approximately 7 per 1,000,000 in neonates and children and 2–5 per 1,000,000 in adults (4). Early management strategies were largely limited to symptomatic treatment, including reduction of intracranial pressure and seizure control. In 1942, Lyons first reported the efficacy of systemic anticoagulation therapy for CVST. Although anticoagulation does not directly dissolve existing thrombi, it can prevent thrombus extension and improve clinical outcomes (8). At present, anticoagulation remains the mainstay of treatment for CVST (4, 9). Randomized controlled trials have confirmed its safety in this setting. Anticoagulation not only reduces mortality and disability but also does not increase the risk of recurrent intracranial hemorrhage, even in patients who initially present with intracranial hemorrhage.

This case report describes a 13-year-old boy who presented with headache. Initial cranial MRI revealed edema in the bilateral frontal and parietal lobes and superior sagittal sinus thrombosis. Given the pronounced signs of intracranial hypertension, mannitol was administered for dehydration after admission. However, as his level of consciousness progressively deteriorated, we performed DSA, followed by mechanical thrombus aspiration of the cerebral venous sinus thrombosis. Postoperatively, the patient's epileptic symptoms improved markedly. Intraoperative exploration revealed bilateral carotid artery occlusion. Subsequent multiregional vascular ultrasound examinations demonstrated atherosclerotic changes in multiple arterial segments. The patient also had high myopia and developed hypertension following this episode. Based on these findings, together with clinical and biochemical tests that excluded other common thrombotic disorders, hyperhomocysteinemia was strongly suspected. To confirm the diagnosis, genetic testing was performed on the patient and both parents.

On postoperative day 4, the patient suddenly developed ventricular fibrillation, suspected to be secondary to pulmonary embolism, which was confirmed by contrast-enhanced Computer Tomography. Given the known association between pulmonary embolism and detachment of lower extremity deep vein thrombosis(DVT), we considered that, although postoperative lower extremity venous ultrasound showed no DVT,iatrogenic vascular puncture during surgery may have caused venous endothelial injury and promoted progressive lower extremity DVT.Initially, we considered LMWH to be a safe and effective option for treating intracranial venous sinus thrombosis in children and preventing lower extremity DVT (10). However, at the time of this event, its anticoagulant effect was clearly inadequate. Subsequent testing revealed markedly reduced antithrombin activity. This likely resulted from homocysteine-induced impairment (endothelial damage, reduced heparin affinity) (11, 12), consumption by active thrombosis, and atherosclerosis-driven suppression of AT-III expression via immune complexes, CD14 + CD64 + monocytes, and TNF-α/IL-6 in endothelial cells (13, 14). Given these mechanisms, the anticoagulant effect of LMWH heparin was considered suboptimal. LMWH heparin exerts its anticoagulant effect primarily by binding to AT-III, which selectively and potently inactivates coagulation factor Xa, thereby inhibiting thrombin generation via an indirect pathway. Its inhibitory activity against thrombin (factor IIa) itself is relatively weak (15). Heparin can also bind directly to platelets and prothrombin, exerting anticoagulant effects through multiple pathways (16), while warfarin achieves anticoagulation by inhibiting hepatic vitamin K epoxide reductase activity (17); both of these agents can circumvent the obstacle of AT-III deficiency.In this case, intravenous urokinase was administered immediately for thrombolysis, followed by unfractionated heparin and then warfarin for ongoing anticoagulation. This strategy was effective: the patient's condition stabilized, consciousness recovered, and no further seizures occurred. This experience underscores an important point: in the presence of severely reduced antithrombin activity, LMWH is not an appropriate choice for anticoagulation. Several case reports have documented the failure of therapeutic-dose LMWH in AT-III deficient patients. Van Bruwaene et al. reported a 12-year-old girl with homozygous AT mutation who presented with undetectable anti-Xa levels despite LMWH therapy, concluding that heparin resistance warrants evaluation for AT deficiency (18). More recently, Kozak et al. described a pregnant patient with AT deficiency who suffered recurrent venous thromboembolism in the 20th week of pregnancy despite standard weight-based LMWH dosing, concluding that standard weight-based LMWH dosing can lead to inadequate anticoagulation in AT-deficient patients (19).

Whole-exome sequencing identified compound heterozygous CBS variants, c.502G > A (p.Val168Met) and c.1111G > A (p.Val371Met). American College of Medical Genetics and Genomics guidelines (20), these were classified as likely pathogenic and pathogenic, respectively, providing a definitive molecular diagnosis of CBS deficiency and highlighting the life-threatening thrombotic risk of unrecognized disease.Both p.V168M and p.V371M were previously listed in ClinVar as variants of uncertain significance (21), reflecting limited clinical and functional data. Our case provides important evidence supporting reclassification. The American College of Medical Genetics and Genomics/Association for Molecular Pathology assessment incorporated PS3_supporting evidence [functional studies showing reduced CBS activity (22)], PM2_supporting evidence [extremely low population frequency (23)], PP3 (consistent deleterious in silico predictions), and, crucially, PM3_strong evidence, as the two variants were confirmed in trans in a patient with a classic CBS-deficient phenotype, consistent with unrelated reported cases (20, 22).This additional evidence supports their pathogenicity and will aid future interpretation of genetic test results.The severe thromboembolic phenotype can be explained by the different locations of the two variants within CBS. p.V168M affects the catalytic domain, where a valine-to-methionine substitution likely disrupts substrate binding or active-site geometry, directly reducing enzyme activity. p.V371M lies in the C-terminal regulatory domain, which modulates enzyme function in response to S-adenosylmethionine (24). This change may impair regulatory sensing or protein stability. We propose that the combination of a catalytic-domain defect and a regulatory-domain defect markedly reduces CBS activity, leading to substantial homocysteine accumulation (25). Elevated homocysteine promotes endothelial dysfunction, oxidative stress, platelet activation, and prothrombotic changes in coagulation (26, 27), creating a systemic prothrombotic state. This mechanism likely underlies the unusual severity and multiplicity of thrombotic events in this adolescent, including cerebral venous sinus thrombosis, arterial occlusions, and pulmonary embolism.

Rozen reported two patients with c.1111G > A (p.Val371Met) homocystinuria in 1994 (28), heterozygous variants at this locus are associated with an increased risk of early-onset atherosclerosis and thromboembolic events (29, 30). Its biochemical phenotype is characterized by markedly reduced CBS enzyme activity, significantly elevated plasma homocysteine and methionine levels, and variable responsiveness to vitamin B6 (31). Given the patient's acute thrombotic presentation, we initiated combination therapy with vitamin B6,folic acidd and betaine, together with warfarin anticoagulation and aspirin as antiplatelet therapy. A low-methionine diet was also recommended. Clinical symptoms and imaging findings showed improvement two weeks after the start of treatment.

## Conclusion

Intracranial venous sinus thrombosis, premature atherosclerosis, hypertension, and acute progressive thromboembolic events should prompt consideration of hyperhomocysteinemia. Although emergency surgery may be beneficial, it carries inherent risks and therefore requires thorough informed consent. Individualized evaluation of patients' biochemical parameters and clinical presentation is essential when selecting anticoagulant therapy. Early genetic testing provides important guidance for both the diagnosis and management of this condition.

## Data Availability

The datasets presented in this study can be found in online repositories. The names of the repository/repositories and accession number(s) can be found in the article/Supplementary Material.
